# Coalescent-Based Species Delimitation Approach Uncovers High Cryptic Diversity in the Cosmopolitan Lichen-Forming Fungal Genus *Protoparmelia* (Lecanorales, Ascomycota)

**DOI:** 10.1371/journal.pone.0124625

**Published:** 2015-05-01

**Authors:** Garima Singh, Francesco Dal Grande, Pradeep K. Divakar, Jürgen Otte, Steven D. Leavitt, Katarzyna Szczepanska, Ana Crespo, Víctor J. Rico, André Aptroot, Marcela Eugenia da Silva Cáceres, H. Thorsten Lumbsch, Imke Schmitt

**Affiliations:** 1 Department of Biological Sciences, Institute of Ecology, Evolution and Diversity, Goethe Universität, Grüneburgplatz 1, 60323, Frankfurt am Main, Germany; 2 Biodiversity and Climate Research Centre (BiK-F), Senckenberg Gesellschaft für Naturforschung, Senckenberganlage 25, 60325, Frankfurt am Main, Germany; 3 Departamento de Biología Vegetal II, Facultad de Farmacia, Universidad Complutense, Plaza de Ramon y Cajal s/n, E-28040, Madrid, Spain; 4 Science & Education, Field Museum of Natural History, 1400 S. Lake Shore Drive, Chicago, IL, 60605, United States of America; 5 Department of Botany and Plant Ecology, Wroclaw University of Environmental and Life Sciences, pl. Grunwaldzki 24a, 50-363, Wroclaw, Poland; 6 Advice Bureau for Bryology and Lichenology Herbarium, Soest, The Netherlands; 7 Universidade Federal de Sergipe, Departamento de Biociências, Itabaiana, Sergipe, Brazil; Consiglio Nazionale delle Ricerche (CNR), ITALY

## Abstract

Species recognition in lichen-forming fungi has been a challenge because of unsettled species concepts, few taxonomically relevant traits, and limitations of traditionally used morphological and chemical characters for identifying closely related species. Here we analyze species diversity in the cosmopolitan genus *Protoparmelia s*.*l*. The ~25 described species in this group occur across diverse habitats from the boreal -arctic/alpine to the tropics, but their relationship to each other remains unexplored. In this study, we inferred the phylogeny of 18 species currently assigned to this genus based on 160 specimens and six markers: mtSSU, nuLSU, ITS, *RPB1*, *MCM7*, and *TSR1*. We assessed the circumscription of species-level lineages in *Protoparmelia s*. *str*. using two coalescent-based species delimitation methods – BP&P and spedeSTEM. Our results suggest the presence of a tropical and an extra-tropical lineage, and eleven previously unrecognized distinct species-level lineages in *Protoparmelia s*. *str*. Several cryptic lineages were discovered as compared to phenotype-based species delimitation. Many of the putative species are supported by geographic evidence.

## Introduction

Lichens are symbiotic organisms consisting of a fungal partner (mycobiont), one or more photosynthetic partners (photobionts; [1]), and diverse bacterial communities [2]. Lichens contribute to ecosystem functioning by nutrient recycling [3], weathering rocks, preventing soil erosion, and acting as pioneer species in barren areas. They inhabit diverse ecosystems from the arctic to the tropics and commonly form an integral part of terrestrial biodiversity [4]. Lichens are preferred model systems for ecological, evolutionary, phylogeographic and population genetic studies of symbiotic associations on the account of their wide, often cosmopolitan, distribution, intriguing eco-physiological interdependence and co-evolutionary and adaptive strategies [5]. Almost one fifth of all known fungi and half of all ascomycetes are lichenized, consisting of approximately 28,000 species worldwide [6,7]. However, studies suggest that the estimate of existing lichen diversity might represent only 50–60% of the real diversity [8,9], as current species recognition in lichen-forming fungi appears to vastly underestimate the true number of species. According to Galloway [8], the number of known taxa in different genera has increased from 20% (*Parmelia sensu stricto*, [10]) to 86% in the New World *Oropogon* [11]. Recent molecular studies have demonstrated the presence of many distinct lineages subsumed under a single species name (e.g., [12–15]). In the basidiolichen fungus *Dictyonema glabratum* a single taxon was found to be composed of at least 126 species [9], thus showing a tremendous amount of unexplored diversity in lichen-forming fungi.

Species recognition in lichen-forming fungi has been a challenge because of i) the few taxonomically relevant characters (reviewed by [16,17]), ii) unsettled species concepts [18,19], iii) and unexplored regions containing high levels of diversity, especially in the tropics [20,21]. Morphological and chemical characters that have commonly been used to circumscribe species may not be useful for identifying closely-related species and often fail to accurately characterize species-level diversity [2,19,22,23]. Accurate species delimitation may be obscured by cryptic speciation [24,25], incongruence between morphology and molecular data [26,27], or incongruence between gene trees and species trees [28]. Moreover, morphological and chemical variations may constitute morpho- or chemotypes of the same species with no molecular differentiation, thus blurring our understanding of species boundaries [29,30]. The implementation of molecular techniques and availability of markers for amplifying phylogenetically informative loci have provided great insights into otherwise unrecognized species complexes. Improved species recognition has important implications for understanding diversity, ecological and biogeographical patterns, factors promoting diversification, and for devising better conservation policies [31].

Different studies have utilized varied combinations of the available techniques for unraveling hidden diversity. For example, Harrington and Near [32] used STEM [33] to explore the independent evolutionary lineages within snubnose darters (*Etheostoma simoterum* species complex). Giarla et al. [15] used two coalescent-based approaches (BP&P and spedeSTEM) for delimiting species in Andean mouse opossums (*Thylamys* spp) using three nuclear loci and found three additional lineages than previously recognized. Leavitt et al. [34,35] used Bayesian population clustering, genealogical concordance, Bayesian species delimitation, and a DNA barcode approach to support the presence of five previously unrecognized species in the lichen-forming fungus *Rhizoplaca melanophthalma* species-complex (Lecanoraceae). Parnmen et al. [36] used a 4-locus phylogenetic approach, combined with GMYC [37,38] and STEM [33] and found at least 12 species in the *Cladia aggregata* complex. Mounting evidence continues to support the perspective that traditional phenotype-based species boundaries fail to adequately characterized species-level diversity in many lichen-forming fungi (reviewed in [22]).

We implemented a molecular approach for species recognition in the cosmopolitan lichen-forming genus *Protoparmelia s*. *str*., combining phylogenetic trees and coalescent-based species delimitation methods. The phylogenetic relationships of the heterogeneous genus *Protoparmelia* have been a matter of debate. Morphological and anatomical characters of this genus show similarity to both Lecanoraceae and Parmeliaceae. *Protoparmelia* was initially placed in Lecanoraceae because it includes crustose lichens, with one-celled hyaline ascospores and *Lecanora*-type ascus [39,40]. Later, secondary metabolite profiles showing the presence of lobaric acid brought into question its placement in Lecanoraceae [41]. Studies on the ascoma ontogeny [42,43] further showed the presence of a typical character of Parmeliaceae in *Protoparmelia*, i.e. cupular exciple, a cup-shaped structure below the hymenium [44]. DNA sequence-based studies suggested *Protoparmelia* to be the sister-group to Parmeliaceae [45–47]. Tropical species of *Protoparmelia* with multispored asci were previously placed in the genus *Maronina* [48]. The authors indicated a close relationship of *Protoparmelia* and *Maronina* on the basis of similar ascus types, and suggested the former to be the multi-spore derivative of *Protoparmelia*. Subsequently, Papong et al. [49] proposed the inclusion of *Maronina* in *Protoparmelia* based on molecular data. However, the tropical clade differs from other species in *Protoparmelia* in being predominantly corticolous, having alectoronic acid as a major compound, and containing many isidiate or sorediate species, whereas most species in the traditional circumscription of *Protoparmelia* are saxicolous and occur in boreal-arctic/alpine and temperate regions.

*Protoparmelia s*.*l*. offers an interesting study system for a variety of reasons. This genus is morphologically and chemically heterogeneous [43,50], and in a previous study [47], we showed that *Protoparmelia s*.*l*. is polyphyletic. In addition, the relationships of most taxa to each other remain largely unexplored. Members of this genus inhabit ecologically diverse habitats, such as boreal-arctic/alpine, temperate, Mediterranean, subtropical, and tropical regions and also vary greatly in their distribution range with some species being cosmopolitan (e.g., *P*. *badia*, *P*. *memnonia*), whereas other, mainly tropical species being locally restricted (e.g., *P*. *orientalis*, *P*. *multifera*). Furthermore, congeners occur on various substrates, with some species growing on bark or decorticated wood, and others on rocks. *Protoparmelia* species exhibit varied life styles. For example, some species are lichenicolous and parasitize other lichen-forming fungi during early parts of their life cycle [50]. Sexual reproduction is common in some species (*P*. *badia* and *P*. *orientalis*), whereas others propagate mainly via asexual propagules (*P*. *isidiata*, *P*. *corallifera* and *P*. *capitata*) with or without any sexual reproduction.

The heterogeneity of characters makes *Protoparmelia s*.*l*. [51] an interesting candidate for testing species delimitation scenarios using multi-locus DNA sequence data. *Protoparmelia s*. *str*. [47] although being a small genus, is sister to the largest family of lichen-forming fungi, i.e., Parmeliaceae [45–47], consisting of approximately 2,800 species distributed in 80 genera [52,53]. Resolving relationships of *Protoparmelia s*. *str*. may contribute to understanding character evolution in an important clade of lichen-forming fungi. The aims of the current study are two-fold: 1) exploring the phylogenetic relationships of *Protoparmelia s*.*l*. species by constructing a multi-locus phylogeny, and 2) assessing the circumscription of lineages in *Protoparmelia s*. *str*. based on multi-locus species-tree inference and coalescent approaches.

## Materials and Methods

### Taxon sampling

This study includes a total of 160 samples of *Protoparmelia s*.*l*. from 18 currently described species. About 70% of the total described species were included in this study. Additionally, three unidentified species, most likely new to science, were also included in the study. We selected 73 taxa from reportedly close relatives of *Protoparmelia s*.*l*. [45,47], namely Parmeliaceae (40 taxa), Lecanoraceae (4 taxa), Gypsoplacaceae ([54]; 2 taxa), *Miriquidica* group (12 taxa), and *Ramboldia* (10 taxa) to infer the relationship of *Protoparmelia s*.*l*. with other taxa within related groups within Lecanorales. Cladoniaceae (5 taxa) were selected as outgroup. Details of the study material and GenBank accession numbers are given in S1 Table.

### DNA amplification and sequencing

Genomic DNA was extracted from lichen thalli using the CTAB method [55]. PCR amplification was performed using general, previously published primers for *RPB1*, *TSR1*, *MCM7*, nuLSU, mtSSU and ITS (Table 1). For some species of *Protoparmelia s*.*l*. and *Miriquidica* group specific primers were designed (Table 1). PCR reactions were carried out in a volume of 25 μl. Each reaction mix contained 2.5 μl buffer, 0.13 μl (0.65 U) Ex Taq polymerase, 1.0 μl dNTP mix (2.5 mM each), 1.0 μl each (10 mM) of the primer set, ca. 20 ng of template, and 16 μl H_2_O. Reactions were performed with the following cycling conditions: initial denaturation at 95°C for 4 min, followed by 35 cycles of 95°C for 30 s, 50°C for 40 s, 72°C for 1 min, and final elongation at 72°C for 5 min. PCR products were checked for amplification on 1% agarose gels. Bands of expected size were extracted using the peqGOLD Gel Extraction Kit. All PCR products were labeled with the Big Dye Terminator v3.1 Cycle Sequencing Kit (Life Technologies, Carlsbad, CA, USA) and cycle sequenced as follows: (1) 1 min 96°C, (2) 26 cycles of 20 s 96°C, 5 s 50°C, and (3) 2 min 60°C. Products were purified using the Big Dye XTerminator Purification Kit (Life Technologies) and then detected on ABI PRISM 3730 DNA Analyzer (Applied Biosystems).

**Table 1:** Primers used in this study.

Taxa	Locus	Primer name	Sequence	Reference
***Protoparmelia***	*RPB1*	fRPB1cR	CNGGCDATNTCRTTRTCCATRTA	[87]
	*RPB1*	gRPB1Af	GADTGTCCDGGDCATTTTGG	[88]
	*RPB1*	RPB1PPsp FOR	GTGCTTTGCTTCAGCAGTGCTC	[47]
	*RPB1*	RPB1PPsp REV	AGCGACGAACATTGCCGTTCGCAC	[47]
	*RPB1*	PPRPB1 FOR	GATGCGGTYTGGCGGCTTTGCAAGCC	This study
	*RPB1*	PPRPB1 REV	GGCTTGCAAAGCCGCCARACCGCATC	This study
	*TSR1*	*120040PP_*TSR1*_FOR	CAGTGTTTTGCCCAGAGAAAGGCTTTCAAG	This study
	*TSR1*	*120082PP_*TSR1*_FOR	TAACGTCCTTGCGAAAGAACGATTAGCGAG	This study
	*MCM7*	MCM7 709 (f)	ACIMGIGTITCVGAYGTHAARCC	[89]
	*MCM7*	MCM7-1348	GAYTTDGCIACICCIGGRTCWCCCAT	[89]
	*MCM7*	PPspecMCM7 FOR	GAICGDTGIGGITRIGARRTITTIC	[47]
	*MCM7*	PPspecMCM7 REV	GIIARRTAITCRTACATGKIRCC	[47]
	*MCM7*	PPMCM7FOR	CTATCGACACGAGCATCCAAG	This study
	*MCM7*	PPMCM7REV	CATGTGACCGRAATGCTTGTATTTC	This study
	nuLSU	LR6 (r)	LR6: CGCCAGTTCTGCTTACC	[90,91]
	nuLSU	AL1R (f)	GGGTCCGAGTTGTAATTTGT	[90,91]
	nuLSU	LR5:	TCCTGAGGGAAACTTCG	[91]
	nuLSU	L3	CCGTGTTTCAAGACGGG	[91]
	nuLSU	LROR	ACCCGCTGAACTTAAGC	[91]
	nuLSU	LSUPPspFOR2	GAAACCCCTTCGACGAGTCGAG	[47]
	nuLSU	LSUPPspREV1	AGATGGTTCGATTAGTCTTTCG	[47]
	ITS	ITS1-F	CTTGGTCATTTAGAGGAAGTAA	[92]
	ITS	ITS2	GCTGCGTTCTTCATCGATGC	[93]
	ITS	ITS3	GCATCGATGAAGAACGCAGC	[93]
	ITS	ITS4	TCCTCCGCTTATTGATATGC	[93]
	ITS	PPITSFFOR1A	GAAGGATCATTATCGAGAGAGG	This study
	ITS	PPITSFREV1A	CTTTCAAAGCGGGAGAAATTTACTAC	This Study
	ITS	PPITSFFOR1Anested	GATCATTATCGAGAGAGGGGCTTC	This Study
	ITS	PPITSFREV1Anested	GGAGAAATTTACTACGCTTAAAG	This Study
	mtSSU	mrSSU1	AGCAGTGAGGGATATTGGTC	[94]
	mtSSU	MSU7:	GTCGAGTTACAGACTACAATCC	[95]
	mtSSU	mrSSU2	CTGACGTTGAAGGACGAAGG	[94]
	mtSSU	mrSSU2R	CCTTCGTCCTTCAACGTCAG	[94]
	mtSSU	mrSSU3R	ATGTGGCACGTCTATAGCCC	[94]
	mtSSU	MSU1	GATGATGGCTCTGATTGAAC	[95]
***Miriquidica***	*RPB1*	RPB1MIRI FOR	CTACAGATGATATCAAGCTCATG	[47]
	*RPB1*	RPB1MIRI REV	CATGAGCTTGATATCATCTGTAG	[47]
	*RPB1*	RPB1MIRIint FOR	CATGACGAAAATCAAGAAACTGCTG	This study
	*RPB1*	RPB1MIRIint REV	CATGCCGTCGCCTATCTCCTTAGTC	Thus study
	*RPB1*	RPB1MIRIFOR1new	TAGCACAACAATCCGGCATTCAAG	This study
	*RPB1*	RPB1MIRIREV1new	TCATTGCTGAGTCCCATGAGCTTG	This study
	*RPB1*	RPB1MIRIREV2new	GCACGAATAATGTCCCCAAGCTTG	This study
	*TSR1*	MIRI_TSR1_FOR	CAACGTTCTGGCTAGAGAGCGTCTGGCAAG	This study
	*TSR1*	*MIRI_40_82_TSR1_REV	CADAGYTGMAGHGYTTGAACCARTTSAC	This study
	*TSR1*	*MIRI_82_TSR1_REV	CAKAGYTGCAGMGCTTTGAACCAGTTGAC	This study
	*TSR1*	TSRMIRIFOR1	TGAGCTGCATCCAAAYGTWCTKGC	This study
	*TSR1*	TSRMIRIINTREV	TAGCGRTYGAATTTGTGGACGTTG	This study
	*TSR1*	TSRMIRIREV1	AACATGTAGCGRAYIGTSACGAG	This study
	*TSR1*	GS1_22TSR1_FOR	GAKCCCATGARCCAGAAGAWTG	This study
	*TSR1*	GS1_22TSR1_REV	GAAGAACATGTASCGGACSGTCAC	This study
	*MCM7*	MCM7MIRI FOR	CAATTTACTCCAATGACTGAATGTC	[47]
	*MCM7*	MCM7MIRI REV	CATGCCGTCGCCTATCTCCTTAGTC	[47]
	nuLSU	NULSUMIRIINT FOR	CTCGGACCGAGGATCGCGCTTC	This study
	nuLSU	NULSUMIRIINT REV	GAAGCGCGATCCTCGGTCCGAG	This study
	nuLSU	NULSUMIRIFOR1	CAGAGACCGATAGCGCACAAGTAGAG	This study
	nuLSU	NULSUMIRIREV1	GAGCCTCCACCAGAGTTTCCTCTG	This study
	ITS	ITSfMIRI FOR	TATCGAGTGGAGGGGCTTCGCTC	[47]
	ITS	ITSfMIRI REV	TAACGTTTAGGCGGTTGTTGGC	[47]
	ITS	ITSFMIRIFOR1	GAATTCAGTGAATCATCGAATCTTTG	This study
	ITS	ITSFMIRIREV1	AGAGTGTAATGACGCTCGAACAGG	This study
*Ramboldia*	*RPB1*	RPB1RAMBINTFOR	GTCTGCCATAATTGYGGCAAGATC	This study
	*RPB1*	RPB1RAMBINTREV	GAYATTTCCACAACCRCCATGATC	This study
	*RPB1*	RPB1RAMFORgroup1	GTYTGCCATAATTGCGGCAAGATC	This study
	*RPB1*	RPB1RAMREVgroup2	ATGTGRCGAAARATRTTKAGSGCC	This study

Taxon specific primers were designed for some *Protoparmelia*, *Ramboldia* and *Miriquidica* species.

For each locus, consensus sequences were assembled separately and aligned using MAFFT [56] as implemented in Geneious v5.4 [57], followed by manual editing. Gaps were treated as missing data and ambiguously aligned nucleotides were excluded.

### Phylogenetic analyses

#### Model selection

Model selection was performed to find the best-fitting model for each data set. We used the Corrected Akaike Information Criterion (AICc) [58] as implemented in jModelTest v2.1.1 [59].

#### Congruence among loci

To test the level of congruence among loci, we used the Congruence Among Distance Matrices test (CADM, [60]), as implemented in the package ape in R. The null hypothesis assumes that all tested phylogenetic trees are completely incongruent. Incongruence here refers to phylogenetic trees with different topologies among loci, which suggests completely distinct evolutionary histories. The level of congruence ranges from 0 to 1. In addition, maximum likelihood (ML) analyses were performed individually on each locus with RAxML-HPC BlackBox v8.1.11 [61] on the Cipres Science gateway [62] using the default GTR + G model with 1,000 bootstrap (BS) replicates. Conflicts were considered significant if individuals grouped in a clade with ≥ 70% BS support in one data set, but in a different clade with high support in another locus.

#### Phylogeny of *Protoparmelia s*.*l*.

Since no supported conflicts were observed in single locus trees and CADM analysis rejected the hypothesis of incongruence among loci, data sets were concatenated (see Results). The maximum likelihood search was performed on the concatenated 6-locus data set including all the relatives of *Protoparmelia s*.*l*. with RAxML-HPC BlackBox v8.1.11 [61] on the Cipres Scientific gateway [62]. Only those taxa for which the sequence information was available for at least three loci were included in the concatenated data set. The default GTR + G model was used as the substitution model and data was partitioned according to the different genes. *RPB1*, *TSR1* and *MCM7* data were also partitioned by codon position.

Bayesian inference was performed using the best fitting model as inferred by jModelTest, for the single as well as concatenated data sets as implemented in MrBayes v3.2.1 [63,64] on the Cipres Scientific gateway [62]. Two parallel MCMCMC runs were performed each using four chains and 5,000,000 generations, sampling trees every 100th generation. A 50% majority rule consensus tree was generated from the combined sampled trees of both runs after discarding the first 25% as burn-in (12,500 trees, likelihoods below stationary level).

#### Phylogeny of *Protoparmelia s*. *str*.

Maximum likelihood analysis was performed individually on each locus of *Protoparmelia s*. *str*. (excluding Lecanoraceae, Parmeliaceae, *Miriquidica* group and *Ramboldia* clades), with RAxML-HPC BlackBox v8.1.11 [61] on the Cipres Science gateway [62], using the default GTR + G model, with 1,000 BS replicates. Gypsoplacaceae was used as outgroup. Only taxa for which sequence information was available for at least three loci were included in the concatenated data set. The default GTR + G model was used as the substitution model and the data was partitioned according to the different genes. For *RPB1*, *TSR1* and *MCM7* data were also partitioned by codon position. Since no supported conflicts were observed in single locus trees and CADM analysis rejected the hypothesis of incongruence among loci, data sets were concatenated. Maximum likelihood search was then performed on the concatenated 6-locus data set using RAxML-HPC BlackBox v8.1.11 [61] on the Cipres Scientific gateway v3.3 [62].

We performed jModelTest for each locus on the reduced data set to select the best locus-specific models of evolution.

Bayesian inference was performed using the best fitting model as suggested by jModelTest, for the single and concatenated data sets separately as implemented in MrBayes v3.2.1 [63,64] on the Cipres Scientific gateway [62]. Two parallel MCMCMC runs were performed each using four chains and 5,000,000 generations, sampling trees every 100th generation. A 50% majority rule consensus tree was generated from the combined sampled trees of both runs after discarding the first 25% as burn-in (12,500 trees).

*BEAST as implemented in BEAST v2.1 [65] was used to estimate the species tree for BP&P [66]. We used a Birth-Death process and gamma-distributed population sizes for the species tree prior and a pairwise linear population size model with a constant root. *BEAST incorporates the coalescent process and the uncertainty associated with gene trees and nucleotide substitution model parameters and estimates the species tree directly from the sequence data. For each locus, the closest model to the best-suggested model from jModelTest under the AICc criterion was selected as the best substitution model for *BEAST. Two independent Markov chain Monte Carlo (MCMC) analyses were performed for a total of 100,000,000 generations, sampling every 5,000 steps. Default values were used for the remaining priors. Convergence of the runs to the same posterior distribution and the adequacy of sampling (using the Effective Sample Size [ESS] diagnostic) were assessed with Tracer v1.4 [67]. After removing the first 20% of the samples as burn-in, all runs were combined to generate posterior probabilities of nodes from the sampled trees using TreeAnnotator v1.7.4 [68]. The species tree produced by *BEAST was subsequently used for inferring speciation probabilities by BP&P [66].

### Species delimitation in *Protoparmelia s*. *str*.

**Fig 1:**
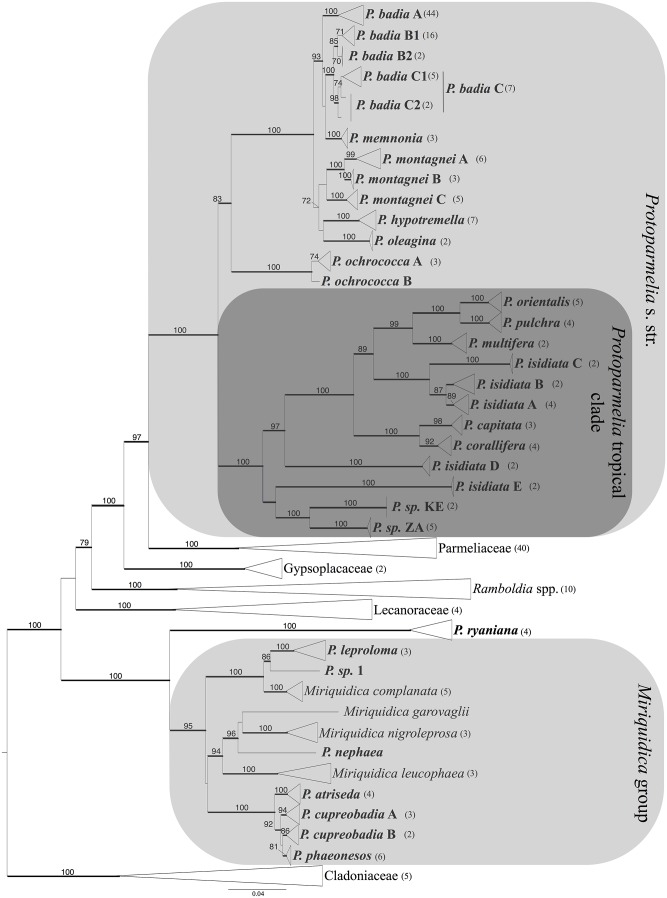
Phylogeny of *Protoparmelia sensu lato* and its allies based on a concatenated 6-locus data set including ITS, nuLSU, mtSSU, *MCM7*, *TSR1* and *RPB1* sequences. This is a maximum likelihood tree. Numbers above branches indicate ML BS ≥ 70%. Branches in bold indicate Bayesian posterior probabilities (PP) ≥ 0.94. Terminal clades were collapsed for clarity of presentation. The length of the triangle corresponds to branch lengths. Numbers in parentheses indicate number of specimens included in collapsed clade. Identity of each specimen in a clade is given in Supporting information S1 Table. *Protoparmelia s*.*l*. species are in bold.

**Fig 2:**
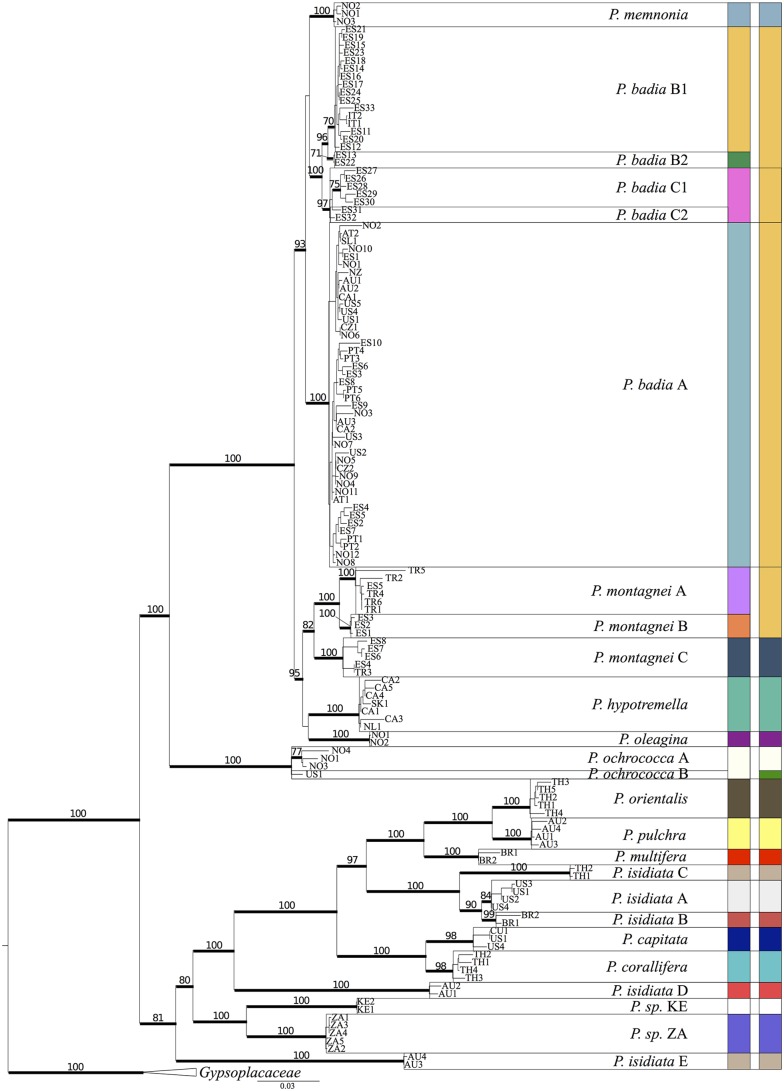
Phylogeny of *Protoparmelia s*. *str*. based on six concatenated loci. Numbers above branches indicate ML BS ≥ 70%. Branches in bold indicate Bayesian posterior probabilities (PP) ≥ 0.94. Specimen indicators include country codes (see Supporting information S1 Table). Taxon names refer to putative species supported by ML BS ≥ 70% or Bayesian Inference (PP ≥ 0.94), and tested for speciation probabilities using BP&P and spedeSTEM. Colored boxes indicate species supported by BP&P (left) and spedeSTEM (right), respectively.

For testing the species boundaries in *Protoparmelia s*. *str*. [47], currently accepted taxa were taken as putative species (12 described species). In addition well-supported (BS ≥ 70%, PP ≥ 0.94) monophyletic clades from ML and Bayesian phylogenies were taken as putative species, resulting in a 25-species scenario (Figs 1 and 2).

The marginal posterior probability of 25-species scenario suggested by molecular data was estimated using the program BP&P v3 [66]. BP&P utilizes reversible-jump Bayesian Markov chain Monte Carlo (MCMC) algorithms for analyzing phylogenetic data from multiple loci to generate speciation probabilities of assigned species. It takes into account uncertainties due to unknown gene trees and ancestral coalescent processes. This method accommodates the species phylogeny as well as incomplete lineage sorting due to ancestral polymorphism. Species tree from *BEAST was used to infer the speciation probabilities by BP&P. BP&P v3 incorporates nearest-neighbor interchange (NNI) algorithm allowing changes in the species tree topology, eliminating the need for a fixed user-specified guide tree [66]. BP&P gives the posterior probability of each delimited species and the posterior probability for the number of delimited species. A gamma prior *G* (1, 10), with mean 1/10 = 0.1 (one difference per 10 bp) was used on the population size parameters (s). The age of the root in the species tree (τ_0_) was assigned the gamma prior *G* (2, 2000) which means 0.1% of sequence divergence, while the other divergence time parameters were assigned the Dirichlet prior [66]. Each analysis was run twice to confirm consistency between runs.

We also used spedeSTEM for calculating probabilities of the species scenario. SpedeSTEM [69] is based on the multilocus species-tree method STEM [33]. It assumes all putative species as separate lineages and estimates gene trees in PAUP* [70]. It then calculates the likelihood for alternative species trees in various permutations and combinations of subpopulations by collapsing two or more species into a single lineage using previously estimated gene trees. Species boundaries are then compared using Akaike information criteria and gives probabilities of different species scenarios. We used θ = 0.05 and each analysis included 500 replicates. We tested all 25 possible permutations for clustering within taxonomic species.

## Results

### DNA sequences

We generated 716 new sequences for this phylogeny, including 142 *RPB1*, 116 *TSR1*, 84 *MCM7*, 150 nuLSU, 127 mtSSU and 107 ITS sequences. The data sets included 310 sequences downloaded from NCBI. A total of 233 taxa were analyzed. The percentage of missing data for each locus was: *RPB1*- 17.17%, *TSR1*- 36.48%; *MCM7*–44.2%, nuLSU- 8.59%, mtSSU—21.45% and ITS- 26.6%.

### Model test

Bayesian analysis on the complete data set and the reduced *Protoparmelia s*. *str*. data set was performed using the best fitting model for each locus in the concatenated sequence as shown in Table 2.

**Table 2:** Genetic characteristics of nuclear loci used in this study.

Full data set			
Locus	No. of seq	length of alignment	Best model
*RPB1*	142	696	012232+G
*TSR1*	196	756	HKY+I+G
*MCM7*	131	655	012212+I+G+F
nuLSU	212	1064	TIM1+I+G
mtSSU	185	834	012212+I+G+F
ITS	168	807	012030+I+G
Concatenated	233	4812	NA
***Protoparmelia s*. *str*.**			
**Locus**		**length of alignment**	**Best model**
*RPB1*	114	696	012232+G+F
*TSR1*	98	754	TPM2uf+G
*MCM7*	63	672	HKY+G
nuLSU	126	972	TIM1+I+G
mtSSU	93	839	: 012212+I+G+F
ITS	96	787	011230+I+G+F
Concatenated 6 loci	138	4720	NA

Genetic characteristics of nuclear loci used in this study, including the total number of sequences per locus, length of the alignment; and best model of evolution selected using the Akaike information criterion as suggested by jModelTest.

For *BEAST, the first available best fitting model for each locus in the concatenated data set, from the models suggested by jModelTest v2.1.1 were the following: *RPB1*: GTR, *TSR1*: HKY, *MCM7*: HKY, nuLSU: GTR, mtSSU: HKY, and ITS: GTR.

### Congruence among loci

CADM results showed no significant incongruence among loci, thus allowing concatenation. The null hypothesis of complete incongruence among loci was rejected for both complete (W = 0.75; p<0.0001) and reduced (W = 0.84; p<0.0001) data sets.

### Phylogeny of *Protoparmelia*

#### *Protoparmelia s.l.*

Nuclear and mitochondrial gene partitions supported the same overall topology. The concatenated six-locus data set contained 233 specimens. Gene partitions had the following lengths: 696 bp for *RPB1*, 756 for *TSR1*, 655 bp for *MCM7*, 1064 bp for nuLSU rDNA, 834 bp for mtSSU and 807 bp for ITS rDNA. The total length of the concatenated alignment was 4812 bp (dryad doi:10.5061/dryad.0q515). The ML tree for the concatenated data set is presented in Fig 1. Nodes with BS ≥ 70% and Bayesian posterior probability (PP) ≥0.94 were considered as supported.

The 6-locus data set yielded a resolved and well-supported topology of *Protoparmelia s*.*l*. (Fig 1). Members of the genus grouped either in *Protoparmelia s*. *str*. [47], or with representatives of the genus *Miriquidica* (“*Miriquidica*-group” in Fig 1), or as sister to the *Miriquidica*-group (*P*. *ryaniana*). The family Parmeliaceae *s*. *str*. formed a well-supported monophyletic group (BS = 100%, PP = 1; Fig 1), which was confirmed to be sister to *Protoparmelia s*. *str*. (BS 97% and PP = 1). Within *Protoparmelia s*. *str*. we found two distinct clades. One contained species with boreal-arctic/alpine, montane, temperate and Mediterranean distributions (*P*. *badia*, *P*. *memnonia*, *P*. *hypotremella*, *P*. *montagnei*, *P*. *oleagina*, *P*. *ochrococca*), the other contained species with subtropical and tropical distributions (*P*. *capitata*, *P*. *corallifera*, *P*. *isidiata*, *P*. *multifera*, *P*. *orientalis*, *P*. *pulchra*, and two yet undescribed species from Kenya and South Africa, respectively).

Six species of *Protoparmelia* (*P*. *atriseda*, *P*. *cupreobadia*, *P*. *leproloma*, *P*. *phaeonesos*, *P*. *ryaniana* and *P*. *sp*. 1) including one yet undescribed species formed a monophyletic group together with *Miriquidica* spp.

#### *Protoparmelia s. str.*

The concatenated six-locus data set contained 138 specimens, including two taxa from outgroup Gypsoplacaceae. Gene partitions had the following lengths: 696 bp for *RPB1*, 754 for *TSR1*, 672 bp for *MCM7*, 972 bp for nuLSU rDNA, 839 bp for mtSSU and 787 bp for ITS rDNA. The total length of the concatenated alignment was 4720 bp. Most species as currently circumscribed were monophyletic, except *P*. *isidiata*, which formed three independent lineages within the tropical clade (*P*. *isidiata* A-C, D and E), and the cosmopolitan species *P*. *badia*, which contained multiple supported lineages and formed a species complex with *P*. *memnonia* (Fig 2). We found evidence for cryptic species-level diversity in the nominal taxa *P*. *badia*, *P*. *montagnei*, and *P*. *isidiata* (clade *P*. *isidiata* A-E). Cryptic diversity corresponded to biogeographic patterns in *P*. *isidiata* (clades A-C representing North America, South America and Asia, respectively). Within *P*. *badia*, the largest lineage (clade *P*. *badia* A) was cosmopolitan, whereas the other supported lineages had a Mediterranean, or Iberian distribution (Fig 2).

### Species delimitation in *Protoparmelia s*. *str*.

**Fig 3:**
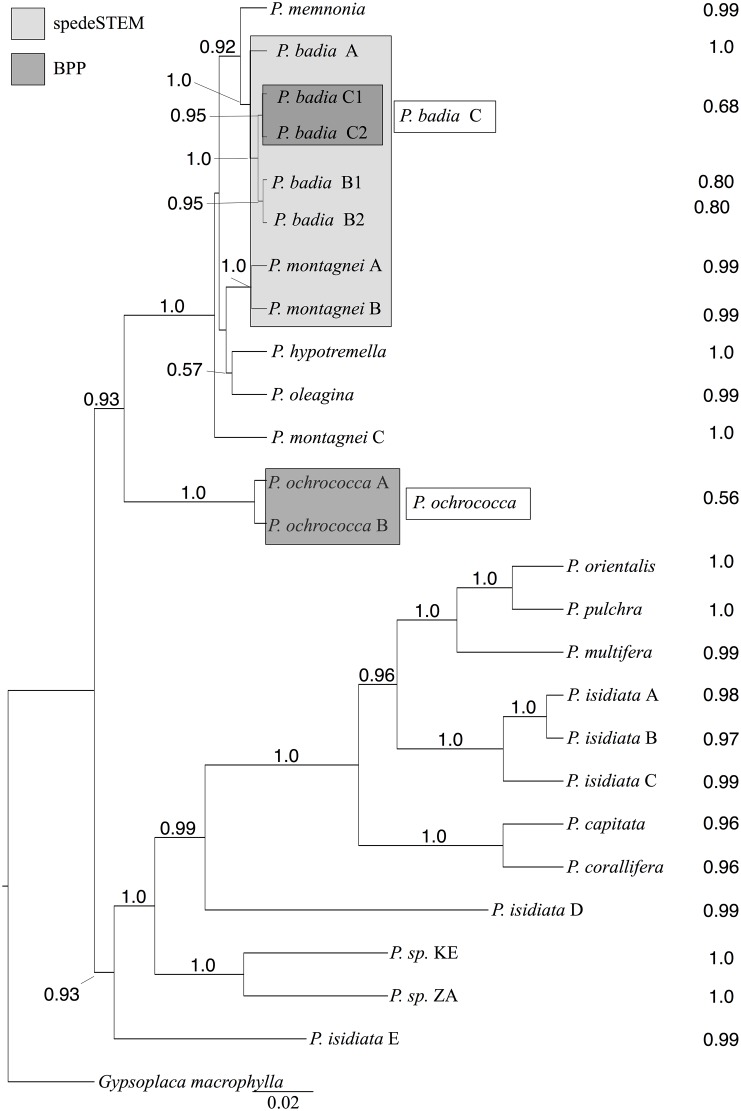
*BEAST species trees for *Protoparmelia s*. *str*. as suggested by ML (BS ≥ 70%) or Bayesian (PP ≥ 0.94). Posterior probabilities at nodes indicate support from the *BEAST analyses. The posterior probability of each delimited species calculated by BP&P are indicated in front of each putative species. Boxes in dark grey indicate clades not supported as separate taxa by BP&P. *Protoparmelia badia* B1 & B2 were supported as separate species whereas *P*. *badia* C1 & C2 were not supported as separate species (referred to as *P*. *badia* C) by BP&P. Box in light grey indicates species not supported as separate taxa by spedeSTEM.

We treated terminal clades supported by ≥ 70% BS and ≥ 0.94 PP (Figs 1 and 2) as putative species for species delimitation analyses. This resulted in a 25-species scenario for *Protoparmelia s*. *str*., in contrast to the current 12-species scenario for *Protoparmelia s*. *str*., based on morphological and chemical characters. The 25-species scenario in *Protoparmelia s*. *str*. was then investigated for species delimitation using BP&P and spedeSTEM. BP&P supported the presence of 23 species with highest probability (PP = 0.41127). Posterior probability of each delimited species is given in Fig 3. *Protoparmelia ochrococca* A & B, *P*. *badia* C1 & C2 were not supported as separate species by BP&P. SpedeSTEM supported 19-species scenario (*P*. *badia* A, *P*. *badia* B1 & B2, *P*. *badia* C1 & C2, *P*. *montagnei* A & B collapsed as one species; Fig 3, θ = 0.05, number of runs = 500), using the model that receives the highest support (100% of the model weighting; Table 3). Sixteen putative species (*P*. *memnonia*, *P*. *hypotremella*, *P*. *oleagina*, *P*. *montagnei* C, *P*. *orientalis*, *P*. *multifera*, *P*. *pulchra*, *P*. *capitata*, *P*. *corallifera*, *P*. *sp*. KE, *P*. *sp*. ZA and the five cryptic isidiate lineages in *P*. *isidiata*) were supported as separate lineages by both BP&P and spedeSTEM (Table 4), therefore we suggest these clades to be evolutionary independent. We found conflicting speciation scenarios for *P*. *ochrococca* A & B, *P*. *badia* A, B1, B2, & C and *P*. *montagnei* A & B by the two species delimitation approaches (Fig 2).

**Table 3:** SpedeSTEM validation results.

Single AIC calculation					
k	ln	AIC	delta	modelLik	wi
1	-242465.0193	484932.0386	187997.2007	0.00	0.00
2	-230753.3543	461510.7085	164575.8707	0.00	0.00
3	-186257.4103	372520.8205	75585.98267	0.00	0.00
4	-184345.6627	368699.3255	71764.48762	0.00	0.00
5	-180035.244	360080.4881	63145.65023	0.00	0.00
6	-175225.9999	350463.9998	53529.162	0.00	0.00
7	-164198.5726	328411.1453	31476.30744	0.00	0.00
8	-160488.1749	320992.3498	24057.51199	0.00	0.00
9	-160402.6815	320823.363	23888.52519	0.00	0.00
10	-154132.577	308285.1541	11350.31624	0.00	0.00
11	-153575.6078	307173.2155	10238.37768	0.00	0.00
12	-153474.4072	306972.8143	10037.97648	0.00	0.00
13	-150731.9074	301489.8148	4554.97696	0.00	0.00
14	-149265.1449	298558.2898	1623.452	0.00	0.00
15	-149048.2275	298126.4551	1191.61724	0.00	0.00
16	-148866.247	297764.4941	829.65624	0.00	0.00
17	-148702.0018	297438.0037	503.16584	0.00	0.00
18	-148652.4701	297340.9402	406.10232	0.00	0.00
**19**	**-148448.4189**	**296934.8378**	**0**	**1.00**	**1.00**
20	-148536.6081	297113.2162	178.37836	0.00	0.00
21	-148526.4393	297094.8785	160.04068	0.00	0.00
22	-148522.9071	297089.8141	154.97628	0.00	0.00
23	-148515.6023	297077.2046	142.36672	0.00	0.00
24	-148515.525	297079.05	144.21212	0.00	0.00
25	-148513.4861	297076.9721	142.13428	0.00	0.00
**Multiple AIC calculation**					
**k**	**ln**	**AIC**	**delta**	**modelLik**	**wi**
1	-242465.0193	484932.0386	187997.2007	0.00	0.00
2	-230753.3543	461510.7085	164575.8707	0.00	0.00
3	-186257.4103	372520.8205	75585.98267	0.00	0.00
4	-184345.6627	368699.3255	71764.48762	0.00	0.00
5	-180035.244	360080.4881	63145.65023	0.00	0.00
6	-175225.9999	350463.9998	53529.162	0.00	0.00
7	-164198.5726	328411.1453	31476.30744	0.00	0.00
8	-160488.1749	320992.3498	24057.51199	0.00	0.00
9	-160402.6815	320823.363	23888.52519	0.00	0.00
10	-154132.577	308285.1541	11350.31624	0.00	0.00
11	-153575.6078	307173.2155	10238.37768	0.00	0.00
12	-153474.4072	306972.8143	10037.97648	0.00	0.00
13	-150731.9074	301489.8148	4554.97696	0.00	0.00
14	-149265.1449	298558.2898	1623.452	0.00	0.00
15	-149048.2275	298126.4551	1191.61724	0.00	0.00
16	-148866.247	297764.4941	829.65624	0.00	0.00
17	-148702.0018	297438.0037	503.16584	0.00	0.00
18	-148652.4701	297340.9402	406.10232	0.00	0.00
**19**	**-148448.4189**	**296934.8378**	**0**	**1.00**	**1.00**
20	-148536.6081	297113.2162	178.37836	0.00	0.00
21	-148526.4393	297094.8785	160.04068	0.00	0.00
22	-148522.9071	297089.8141	154.97628	0.00	0.00
23	-148515.6023	297077.2046	142.36672	0.00	0.00
24	-148515.525	297079.05	144.21212	0.00	0.00
25	-148513.4861	297076.9721	142.13428	0.00	0.00

spedeSTEM validation results, using θ = 0.5. The absolute difference between the AICc score for the given model and the best-fitting one is listed under the column labeled ‘‘Di” and the model weighting is listed under the column labeled “wi”.

**Table 4:** Summary of results of ML, Bayesian and species delimitation analyses (BP&P and spedeSTEM).

Putative species	BS	BS1	PP	PP1	BP&P	spedeSTEM
*Protoparmelia badia* A	100		1		1.0	-
*Protoparmelia badia* B1	71	85	0.61	1	0.80	-
*Protoparmelia badia* B2	70		—		0.80	-
*Protoparmelia badia* C1	74	98	0.97	1	0.68	-
*Protoparmelia badia* C2	98		—			-
*Protoparmelia capitata*	98		1		0.96	+
*Protoparmelia corallifera*	92		1		0.96	+
*Protoparmelia hypotremella*	100		1		1.0	+
*Protoparmelia isidiata* A	89		0.96		0.98	+
*Protoparmelia isidiata* B	64		1		0.97	+
*Protoparmelia isidiata* C	100		1		0.99	+
*Protoparmelia isidiata* D	100		1		0.99	+
*Protoparmelia isidiata* E	100		1		0.99	+
*Protoparmelia memnonia*	100		1		0.99	+
*Protoparmelia montagnei* A	99		1		0.99	-
*Protoparmelia montagnei* B	100		1		1.0	-
*Protoparmelia montagnei* C	100		1		1.0	+
*Protoparmelia multifera*	100		1		1.0	+
*Protoparmelia ochrococca* A	74		0.82	1	0.56	+
*Protoparmelia ochrococca* B	NA		NA			+
*Protoparmelia oleagina*	100		1		0.99	+
*Protoparmelia orientalis*	100		1		1.0	+
*Protoparmelia pulchra*	100		1		1.0	+
*Protoparmelia sp*. KE	100		1		1.0	+
*Protoparmelia sp*. ZA	100		1		1.0	+

Clades in Column A represent putative species having ML BS support ≥ 70% or Bayesian PP ≥ 0.94, tested for speciation probabilities using BP&P and spedeSTEM. + represents supported clades;—represents clades not supported. Clades supported by BP&P were considered as separate species. ^1^ represents support for 22-species scenario (*P*. *badia* B1, B2 and *P*. *badia* C1, C2, *P*. *ochrococca* A, B collapsed), i.e. three instead of five putative species within *Protoparmelia badia*.

## Discussion

The genus *Protoparmelia* is more diverse than the traditional taxonomy suggests. This diversity comprises several previously undescribed species, and cryptic lineages within currently accepted species. Most species of *Protoparmelia* belong to *Protoparmelia s*. *str*., consisting of a tropical and an extra-tropical clade. The tropical clade includes several taxa having multispored asci, which were formerly classified in the genus *Maronina* [48,49,71,72]. All of its members, except the undescribed South African and Keniyan species, are corticolous. Most members of the tropical clade reproduce vegetatively, although a limited number of species propagate predominantly via sexual reproduction. All supported genetic species-level lineages in the tropical clade are congruent with biogeographic origin of the specimens. Evolutionary rates, i.e. rates of base substitutions in an evolutionary lineage over time, appeared to be accelerated in the tropical clade. This phenomenon has also been previously observed in tropical lichens, and attributed to shorter generation times, higher metabolic rates, continuous physiological activity of a poikilohydric organism in a moist environment, and lack of sexuality [73]. The extra-tropical clade contains mostly saxicolous taxa, most of which reproduce sexually. Within this group, while some species show restricted distribution, some other have wide geographic distributions, such as the cosmopolitan *P*. ‘*badia* A’ and *P*. *hypotremella* which occurs in Europe and North America. Five previously described species and one species putatively new to science group with members of the genus *Miriquidica*. In contrast to members of *Protoparmelia s*. *str*., which produce lobaric or alectoronic acids, these taxa synthesize norstictic acid as major secondary metabolite. Many of these species parasitize other lichens during at least parts of their life cycle [50], a lifestyle not known from members of *Protoparmelia s*. *str*. Close affiliations between *Miriquidica* and *Protoparmelia* based on shared morphological characteristics have been suggested before [74,75], and a recent molecular study confirmed the close relationship of the *P*. *atriseda*-group and *Miriquidica* [47]. A revision of the genus *Miriquidica* based on molecular data is currently under way by our colleagues (Timdal, pers. comm.).

### Speciation analyses and cryptic diversity

We validated the 25-species scenario for *Protoparmelia s*. *str*., which was based on the previously defined species and a few new clades suggested by molecular data (phylogenetic species concept). Based on our sampling, this study largely supported traditionally circumscribed *Protoparmelia s*. *str*. species as distinct lineages. However, exceptions included *P*. *isidiata*, an asexual tropical species, and *P*. *badia*, a sexually reproducing, boreal-arctic/alpine cosmopolitan species. The former was found to be polyphyletic and separated into three distinct lineages, while the later was paraphyletic and formed a species complex with *P*. *memnonia* (Figs 1 and 2).

The combined use of species-tree topology and coalescent methods revealed the presence of several cryptic lineages in *Protoparmelia s*. *str*. This is in concordance with other studies in which molecular markers in combination with statistical tools revealed many genetically distinct lineages hidden under a single taxon [9,36,76–78]. Studies suggest that cosmopolitan species such as *P*. *badia* may reveal high cryptic diversity [79,80], which may or may not correlate to geography. In our study we found that the cosmopolitan *P*. *badia* as currently delimited consists of at least four independent evolutionary lineages. Among these newly recognized lineages only *P*. *badia* A turns out to be cosmopolitan, inhabiting boreal-arctic/alpine habitats in North America, Europe, New Zealand and Australia. The other lineages of *P*. *badia* (*P*. *badia* B1, B2 and C) have a more limited distribution, having been collected so far on siliceous substrates in Spain and Italy. Cryptic lineages within *P*. *isidiata* (clades A-C) also correspond to broad biogeographic patterns, while lineages identified within *P*. *montagnei* co-occur in the Mediterranean region (Fig 2). Thus, geographic evidence supports species delimitation suggested by coalescent-based speciation analyses in most cases. However, current sampling in many lineages is relatively sparse and does not allow conclusions about finer-scale biogeographic patterns, such as endemism. It remains to be seen whether sympatrically-occurring cryptic lineages identified in this study are supported by additional, previously overlooked morphological or chemical characteristics. We have preliminary evidence that the currently recognized *P*. *montagnei* chemotypes [81] correspond to the three molecular clades and may thus indeed represent closely related, but separate species.

Conflicts between different methodological approaches to species delimitation are common [13,15,78,82]. In general we follow the approach of adopting the speciation scenario that is supported by both the analyses, in our case 16 species [83]. For some clades, i.e. *P*. *ochrococca* A & B, *P*. *badia* A, B1, B2 & C, *P*. *montagnei* A & B, the most likely speciation scenario given by spedeSTEM deviates from BP&P, and contradicts supported branching patterns in the phylogeny (Figs 2 and 3). For *P*. *badia* A, B1, B2 & C, *P*. *montagnei* A & B phylogenetic tree and BP&P supported these clades to be evolutionary independent, whereas spedeSTEM suggested them to be a single species. For *P*. *ochrococca* A & B phylogenetic tree and spedeSTEM supported these clades to be evolutionary independent, whereas BP&P suggested them to be a single species. Recent studies indicated that spedeSTEM may be less accurate than other species delimitation methods in cases of recent speciation events [84]. For the clades supported by BP&P and not spedeSTEM, we preferred BP&P results as BP&P has been shown to perform well even when putative species were modeled to have diverged from one another only very recently [84]. In addition, BP&P has been shown to outperform other coalescent-based species delimitation approaches especially when using multi-locus DNA sequence data and a modest number of individuals per species [69,83]. Previously the reliability of BP&P has been suggested to be dependent on the accuracy of the user-provided guide tree. However, in the latest version of BP&P the authors addressed this issue and applied the NNI algorithm, which allows flexibility in the species tree. Moreover BP&P is suggested to be conservative in delimiting species, with high probability to be a reliable indicator of evolutionary independence of the lineages [66]. Therefore in case of conflicts we considered BP&P to be more accurate and suggested the lineages supported by BP&P as distinct species.

Our analyses suggest that the sampled specimens of the tropical *Protoparmelia s*. *str*. group belong to five distinct species. Two sexually reproducing (apotheciate) species, *P*. *multifera* and *P*. *orientalis*, traditionally distinguished by having different minor secondary metabolites [49] were supported as different species and were not sister to each other. In fact, the sexually reproducing species *P*. *pulchra* was sister to *P*. *orientalis*. In addition, we found four distinct asexually reproducing (isidiate) species of *Protoparmelia s*. *str*. Two of these species (*P*. ‘*isidiata* D’ and *P*. ‘*isidiata* E’) occur sympatrically in Australia. Several studies have shown the occurrence of phylogenetically unrelated but morphologically similar lineages thus indicating the presence of high hidden diversity in lichen-forming fungi [25,27,34,85,86].

## Conclusions

Our analyses support the presence of 23 distinct lineages in *Protoparmelia s*. *str*. in contrast to 12 currently delimited species, revealing much more diversity than currently suggested for this genus. Our study shows that the sister group of the largest family of lichen-forming fungi may harbor a considerable amount of cryptic lineages which can be identified using molecular data. These data highlight the presence of substantial phylogenetic diversity especially in the tropics, and the need for careful re-evaluation of morphological and chemical characters in the group.

## Supporting Information

S1 Table Specimens used in this study including voucher information and GenBank accession numbers. (XLSX)
